# Targeting immunometabolism in cancer through pharmacological and lifestyle interventions

**DOI:** 10.1371/journal.pbio.3003617

**Published:** 2026-01-20

**Authors:** Rachael Julia Yuenyinn Tan, Yalei Liu, Weixin Chen, Yuteng Liang, Guang Sheng Ling

**Affiliations:** 1 School of Biomedical Sciences, Li Ka Shing Faculty of Medicine, The University of Hong Kong, Hong Kong SAR, China; 2 Department of Medicine, School of Clinical Medicine, Li Ka Shing Faculty of Medicine, The University of Hong Kong, Queen Mary’s Hospital, Hong Kong SAR, China; University of Birmingham, UNITED KINGDOM OF GREAT BRITAIN AND NORTHERN IRELAND

## Abstract

Immunometabolism, a fundamental biogenic process that supports the function of immune cells, is often disrupted in diseases such as cancer. Tackling metabolic dysregulation at a cellular level has therefore emerged as a focus in drug development. However, as cellular metabolic rewiring takes place in response to both intrinsic factors, which can be targeted pharmacologically, and environmental changes, which cannot, fostering a homeostatic systemic metabolism through diet, exercise, and stress management is essential to support and sustain cellular fitness. This Essay conceptualizes immunometabolism as a process that can be regulated intrinsically and extrinsically and explores the potential for incorporating lifestyle changes and drug therapies that target immunometabolism into treatments for cancer.

## Abstract 2

Immunometabolism is often disrupted in diseases such as cancer, suggesting it could be useful target for therapy. This Essay explores the potential for incorporating both lifestyle changes and drug therapies that target immunometabolism into treatments for cancer.

## Introduction

Among cellular processes, metabolism stands at the core, supplying energy for cell survival, differentiation, proliferation, and functional demands. Likewise, in immune cells, metabolic governance, termed immunometabolism (Box 1), is finely tuned to support their cellular processes. Disruption of immunometabolism is a feature of various diseases. Take cancer, for instance: while it is not commonly defined as a metabolic disease, it can be seen as a consequence of metabolic aberrations, and it causes significant disruption to both systemic metabolism [1–3] and the metabolism of tumor-infiltrating leukocytes [4,5]. This cascade of metabolic alterations further fuels tumor progression and contributes to immunotherapy resistance by impairing anti-tumor responses [1,6,7].

**Box 1 Glossary**

**Fatty acid oxidation**

The breakdown of fatty acids in the mitochondria to generate energy.

**Glycolysis**

The breakdown of glucose into pyruvate to generate energy.

**Immunometabolism**

A series of biochemical reactions in immune cells governing energy production and usage to support their fundamental functions.

**NETosis**

Neutrophil activation-induced form of cell death whereby DNA, histones, and cytoplasmic proteins are released in a ROS-dependent manner.

**Prebiotics**

Factors supporting the activity of beneficial microbes in the body.

**Probiotics**

Living microorganisms which benefit the health and balance of the microbiome in the body.

**Senotherapeutics**

Therapeutic approach targeting senescent cells to suppress their activity.

**Synbiotic**

A combination of live microorganisms and substrate(s) used by them that confers a health benefit to the body.

**T cell exhaustion**

A state of dysfunction in T cells that occurs upon prolonged antigenic stimulation, resulting in inefficient effector activity.

The crosstalk between immunometabolism and circulating metabolites is bidirectional and complex, whereby the composition of the circulating metabolites will affect the way immune cells sense and process environmental cues, and in turn, their responses may act on proximate tissubox_para_fes to alter systemic metabolism [8]. This interplay is especially apparent in the immune cells responsible for tissue homeostasis beyond immunity, such as tissue-resident macrophages (TRMs) and matrix-modulating neutrophils. In response to antigenic challenge, such as infection or neoplasm, immunometabolism within the adaptive immune system is also critical, as a metabolic shift is required for immune activation. Importantly, lifestyle and diet are inevitable regulators of systemic metabolism, as dietary intake and exercise effectively alter circulating metabolite composition.

In this Essay, we discuss factors affecting immunometabolism in health and disease, particularly in cancer, and lifestyle interventions that could potentially improve disease management from a dual systemic and cellular angle. We focus on the importance of metabolic fitness of T cells, macrophages, and neutrophils in improving cancer management, as cancer progression is tightly associated with dysregulation of systemic and immune metabolism.

## Metabolism as a regulator of immune cell plasticity

Cellular metabolism critically regulates immune cell plasticity and function. Key pathways include glycolysis (Box 1), the tricarboxylic acid (TCA) cycle, the pentose phosphate pathway (PPP), fatty acid oxidation (FAO; Box 1), fatty acid synthesis, and amino acid metabolism [9]. These are coordinated by upstream signaling and transcription factors, with mitochondrial activity essential for energy production and signal transduction.

Immune cells exhibit intricate metabolic activities, regulating their fate determination, survival, and function. For example, naive T cells are reliant on oxidative phosphorylation (OXPHOS) to maintain quiescence, while their activation shifts metabolically to aerobic glycolysis upon T cell receptor and CD28 stimulation to support differentiation and proliferation via the ERK–MAPK pathway, calcium influx, and the PI3K–AKT–mTOR axis [10–12]. Different stages of T cell differentiation demand different levels of bioenergy and thus different metabolic programs. For instance, effector T cells are highly glycolytic to provide the burst of energy needed to support cytokine secretion and cytotoxic functions, whereas memory T cells employ FAO and OXPHOS via cell-intrinsic lipolysis to provide a steady stream of energy [13]. Conversely, FOXP3 suppresses glycolysis in regulatory T cells to preserve their suppressive role through the PI3K–AKT pathway [14]. In addition, persistent antigen exposure in the tumor microenvironment (TME) may induce T cell exhaustion (Box 1); through PD-1 signaling activation, glucose metabolism is reduced in favor of FAO and reactive oxygen species (ROS) production, ultimately leading to T cell dysfunction [11,15].

In myeloid cells, macrophage polarization is metabolically regulated, whereby differentiation of pro-inflammatory macrophages depends on glycolysis over the TCA cycle and OXPHOS, whereas differentiation of anti-inflammatory macrophages relies mainly on OXPHOS [16,17]. Macrophages are typically diverse in ontogeny and tissue homeostatic function. For TRMs such as adipose tissue macrophages (ATMs), respiration is shifted from OXPHOS and FAO to glycolysis upon obesity-driven activation via full-length oxidized phospholipid sensing, resulting in low-grade inflammation [18]. By contrast, alveolar macrophages, which facilitate housekeeping of lipid-rich surfactants, employ lipid catabolism to sustain their functions via the GM-CSF–PPARγ axis once activated [19]. In neoplasms, tumor-associated macrophages (TAMs), especially those derived from tumor-infiltrating monocytes, are typically anti-inflammatory. In TAMs, HIF-1α, mTOR, and NF-κB activation promotes both glycolysis and OXPHOS to sustain their immunosuppressive functions [17].

As for neutrophils, oxidative burst is required for most of their functions, and this process is highly glycolytic; neutrophils utilize the glycerol 3-phosphate pathway to shuttle glycolytic byproducts and NADH electrons and maintain their mitochondrial membrane potential to support ROS production [20]. The metabolic plasticity of a cell defines its adaptability to environmental changes, and neutrophils are highly flexible in shifting their metabolic needs. In the TME, where glucose availability is scarce and conditions are highly hypoxic, tumor-associated neutrophils (TANs) dynamically switch to glutamine oxidation and FAO to provide their energetic requirements for continuous ROS production [21]; moreover, the hypoxic TME favors HIF-1α stabilization for PPP activation in neutrophils, increasing the formation of neutrophil extracellular traps (NETs) and facilitating tumor metastasis [22].

In summary, immunometabolic plasticity is important for immune cell survival in response to various environmental cues; however, this flexibility may also equip immune cells to be reprogrammed along undesired trajectories. Given the differential metabolic profiles of leukocytes in homeostasis and disease, it is crucial to think about disease states from an immunometabolic perspective.

## Systemic factors affecting immunometabolism

**Fig 1:**
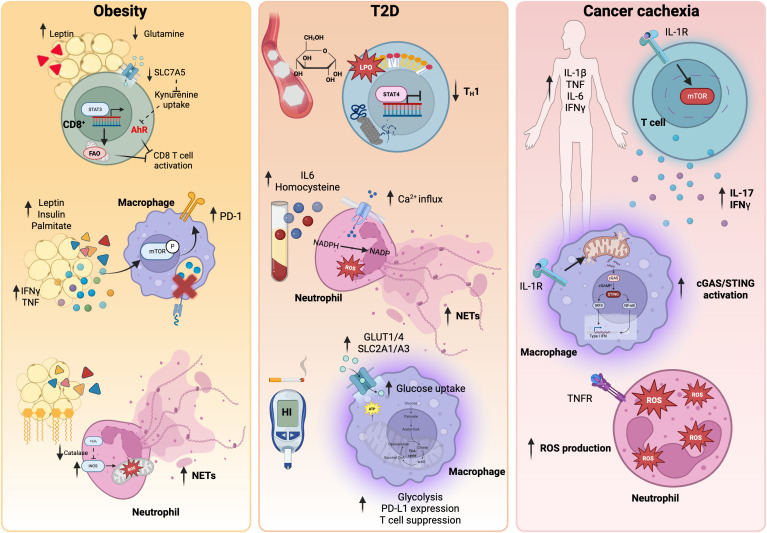
Systemic metabolic diseases that influence immunometabolism. Metabolic diseases can induce immune dysfunction and ultimately affect anti-tumor immunity via immunometabolic deregulation. Obesity, characterized by high adiposity, creates a tissue reservoir for adipokines to trigger immunometabolic rewiring. In CD8^+^ T cells, increased extracellular leptin hampers glutamine and kynurenine uptake via SLC7A5 inactivation, resulting in a metabolic shift towards fatty acid oxidation and repressed AhR activation, ultimately affecting T cell activation upon antigen exposure. In macrophages, secretion of pro-inflammatory adipokines and cytokines effectively triggers PD-1 expression via mTOR activation, resulting in compromised phagocytosis and antigen presentation capability. In neutrophils, the increased uptake of fatty acids restricts catalase activity, resulting in increased inducible nitric oxide synthase and subsequent ROS-facilitated NET release. In type 2 diabetes, T_H_1 cell differentiation is dampened by decreased STAT4 stability due to LPO-induced STAT4 carbonylation. In neutrophils, hyperglycemia-induced high serum homocysteine and IL-6 enables increased calcium influx, which accelerates NADPH-induced ROS and subsequent NETosis. In macrophages, tobacco-induced hyperglycemia activates glucose transporters GLUT 1 and GLUT 4 to enhance glucose uptake and fuels glycolysis, resulting in increased PD-L1 expression and strong T cell suppression. Cancer cachexia, characterized by body wasting and anorexia, causes an increased in circulating IL-1β, TNF, IL-6, and IFNγ. In T cells, IL-1β binding to IL-1R activates mTOR signaling and IL-17 and IFNγ production. In macrophages, IL-1β disrupts mitochondrial membrane potential and causes mitochondrial rupture, resulting in release of mitochondrial DNA and activation of the cGAS–STING pathway. In neutrophils, TNF effectively induces ROS production and accumulation to allow the neutrophil oxidative burst and pro-inflammatory activation. Taken together, these changes result in sustained systemic inflammation. *Created in BioRender. Tan, R. (2026)* *https://BioRender.com/1yes5bx*.

**Fig 2:**
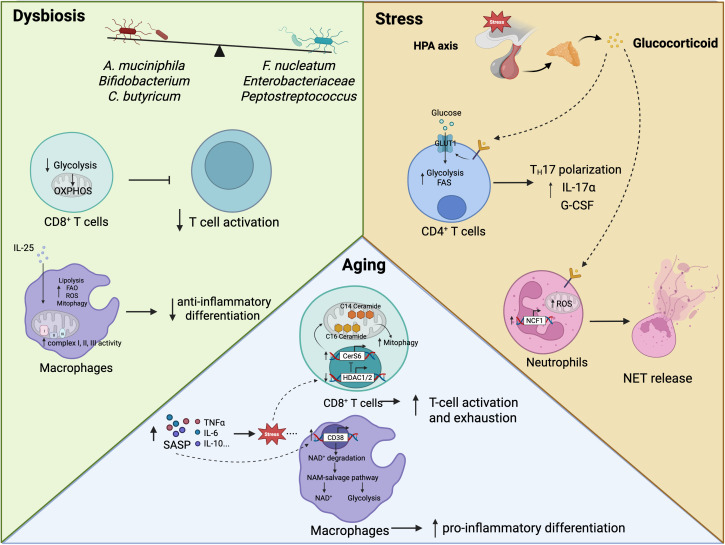
Systemic factors that affect immunometabolism. Various factors can alter systemic metabolism. In cancer, gut dysbiosis gives rise to attenuated anti-cancer immunity. In CD8^+^ T cells, dysbiosis shifts glycolysis towards OXPHOS to attenuate T cell activation. Dysbiosis also causes increased circulating IL-25, which further activates complexes I, II, and III in the electron transport chain in macrophages, leading to increased FAO, ROS production, mitophagy, and ultimately the polarization of macrophages towards an anti-inflammatory phenotype. Psychophysiological stress, indicated by increased glucocorticoid secretion via the HPA axis and glucocorticoid receptor signaling, activates GLUT4 activity and glucose uptake in CD4^+^ T cells to enhance glycolysis and fatty acid synthesis, resulting in T_H_17 cell polarization and subsequent pro-inflammatory IL-17 and G-CSF secretion. Glucocorticoid receptor activation in neutrophils also triggers NCF1 to enhance ROS-induced NET release. Both of these changes create systemic inflammation and anti-tumor immunity. Aging increases SASP and TNF, IL-6, and IL-10 production. Aging drives mitochondrial ceramide accumulation and mitophagy via HDAC1/2-inhibited CerS6 activity, ultimately leading to T cell activation and exhaustion. In macrophages, SASP activates the NAM salvage pathway via upregulated transcription of CD38, in turn expanding the NAD^+^ pool and glycolysis to support pro-inflammatory differentiation. *Created in BioRender. Tan, R. (2026)* *https://BioRender.com/w60jjpa*.

The metabolic functions of immune cells are intricately regulated by their surrounding environment. Systemic factors, such as nutrient availability, chronic inflammation driven by obesity and aging, and stress hormone levels, can disrupt immune cell metabolism, ultimately impairing immune responses (Figs 1 and 2).

### Metabolic diseases

Systemic metabolism unequivocally regulates immunometabolism in physiology and in disease [6]; hence, it is natural for metabolic diseases to alter the immunometabolic milieu (Fig 1). Two of the most concerning metabolic diseases, obesity and type 2 diabetes (T2D), projected to affect 50% [23] and 9.8% [24] of the global population by 2050, respectively, undoubtedly impose strains on immune fitness and increase the risk of co-morbidities. Crucially, both conditions have been linked to increased cancer prevalence and poor prognosis for immune checkpoint blockade (ICB) therapy, underscoring their detrimental impact on anti-tumor immunity [25,26]. In an MC38 mouse model of colorectal cancer, mice with diet-induced obesity exhibited low serum levels of glutamine, which resulted in diminished SLC7A5 transporter activation and a subsequent reduced uptake of kynurenine by CD8^+^ T cells, ultimately dampening their activation [27]. Additionally, leptin-driven adiposity in mouse models of obesity promotes STAT3-facilitated FAO in intra-tumoral CD8^+^ T cells at the expense of glycolytic capability [28]; although this FAO shift is crucial for the formation of memory CD8^+^ T cells [29], the activation process is already compromised by diminished glycolysis, resulting in redundancy as FAO cannot sustain the energetic needs required for effector functions.

In addition to lymphocytes, the myeloid compartment is also closely associated with obesity. ATMs are active recipients of white adipocyte-derived mitochondria, facilitating the clearance of damaged mitochondria, while also adapting to a TCA cycle and OXPHOS-driven metabolic program to maintain their tissue regulatory phenotype [30]. However, this mitochondrial transfer is compromised in obesity, resulting in a glycolytic shift and promoting low-grade inflammation [31]. In mouse TAMs, obesity-driven mTOR activation upregulates PD-1 expression which, in a negative feedback manner, suppresses phagocytosis and antigen presentation [32]. This cascade ultimately impairs tumor immune surveillance in mice with diet-induced obesity. Notably, macrophages are capable of dictating adipogenesis depending on their site of residency within tissue (for example, septal ATMs are pro-adipogenic via TGFβ signaling), suggesting that different microenvironments foster macrophages with different behaviors [33]. Taken together, obesity paves the way for a feedforward loop that drives macrophage metabolic reprogramming, either locally in adipocytes or distally (e.g., in tumors), while in turn, macrophages govern the progression of obesity. In mouse neutrophils, obesity downregulates intracellular catalase and increases iNOS activities, which synergistically enhance the neutrophil oxidative burst and NETosis (Box 1), resulting in increased oxidative toxicity to vasculature, in turn facilitating tumor metastasis [34]. Given that macrophages and neutrophils both have essential roles in tissue homeostasis, it is important for individuals to maintain a healthy weight to minimize the risk of metabolic deregulation in various cell types, which could aggravate clinical manifestations and affect treatment outcomes during disease.

In T2D, peripheral CD8⁺ T cells from patients exhibit reduced basal respiration and glycolysis compared to those from healthy individuals, impairing antigen-specific responses and cytokine production [35]. Although obesity is strongly linked to T2D and may contribute to impaired CD8⁺ T cell immunity, hyperglycemia (a defining feature of T2D) is likely the principal factor driving dysregulated T cell metabolism and function in this condition. Stratifying patients with T2D by glycated hemoglobin (HbA1c) levels reveals that CD4⁺ T cells from individuals with poorly controlled glycemia (HbA1c>8) have impaired T helper (T_H_) 1 cell polarization and functionality despite unaltered T cell receptor (TCR) signaling. This defect is thought to be the result of T cell maladaptation to the hyperglycemic environment, which disrupts mitochondrial homeostasis and triggers lipid peroxidation (LPO); LPO permits the carbonylation and subsequent degradation of STAT4, a transcription factor essential for T_H_1 cell lineage commitment, ultimately impairing CD4^+^ T cell responses [36]. In addition, hyperglycemia impairs CD8⁺ T cell responses, as evidenced by reduced persistence and heightened exhaustion within the TME. Hyperglycemia also enforces excessive glycolytic activity in CAR-T and TCR-T cells, blocking the metabolic shift from glycolysis to FAO and thereby diminishing their retention in tumors [37].

In cancer, hyperglycemia diminishes the beneficial roles of neutrophils while amplifying their pro-tumorigenic effects. Similarly, in T2D, raised homocysteine and IL-6 levels trigger NET formation through both NADPH oxidase-dependent (ROS-mediated) and independent mechanisms [38]. This process leads to vascular injury and may thereby contribute to the increased metastasis and poor prognosis observed in both mouse models of diabetes and in patients with T2D [22]. Interestingly, cigarette smoking-induced hyperglycemia provides another mechanism linking metabolic dysfunction to macrophage functionality. Nicotine-derived nitrosamine ketone (NNK) and benzo[a]pyrene (BaP) exposure shifts macrophage polarization to an anti-inflammatory phenotype in mice fed a high-carbohydrate diet. Mechanistically, BaP–aryl hydrocarbon receptor (AhR)- and NNK–nAChR-mediated signaling upregulates SLC2A1/ALC2A3 and GLUT1/GLUT3 translocation, enhancing glucose uptake and glycolysis in macrophages. This metabolic shift increases IGF2 secretion, which induces PD-L1 expression on lung cancer cells via the IGF2–IR pathway to suppress T cell responses, resultantly facilitating tumor progression [39].

Although cancer is traditionally described as a neoplastic disease, it can also be seen as an unorthodox metabolic disorder, as exemplified by cancer cachexia. As cancer cells are highly anabolic, nutrients are channeled towards them to fuel their proliferative needs, resulting in fat, muscle, and skeletal cell wasting [40]. The most common features of cachexia include increased levels of circulating cytokines such as TNF, IL-1β, IL-6, and IFNγ [41]. While most studies have focused on the effect of cachexia on musculoskeletal homeostasis, its effects on immune cells are equally important. Notably, circulating IL-1β induces a shift in CD4^+^ T cell polarization towards a pro-inflammatory phenotype via mTOR activation, characterized by enhanced IL-17 and IFNγ production [42]. Furthermore, exogenous IL-1β binding to IL-1R in myeloid cells triggers the deterioration of mitochondrial membrane potential and the subsequent release of mitochondrial DNA, promoting a cross-activating event and macrophage response via the cGAS–STING–IRF3 axis [43]. In addition, circulating TNF can trigger neutrophil activation and intracellular ROS production [44]. These immune cell effects provide evidence for the role of cachexia in advancing chronic inflammation and suggest that it may aggravate it in a feedforward manner.

### Gut microbiome dysbiosis

Dysbiosis, an imbalance in gut microbiota composition, is linked to chronic metabolic diseases, inflammation, and aging. It is characterized by reduced microbial diversity, loss of beneficial commensals, and expansion of pathogenic microbes [45]. In particular, the blooming of bacteria in the *Enterobacteriaceae* family often correlates with inflammatory diseases and cancer [46]. A general increase in *Fusobacterium nucleatum*, *Peptostreptococcus anaerobius*, and *P. stomatis*, along with a decrease in *Akkermansia muciniphila*, *Bifidobacterium*, *Clostridium*, and *Butyricum* has been linked to cancer progression and ICB resistance [45,47]. As the gut microbiota is diverse and dynamically influenced by systemic and epigenetic factors, it is difficult to define a healthy microbiota just by characterizing the colonizing microbial species; nevertheless, the dominance of certain microbiome species is still a useful indication of disease susceptibility or prognosis. Among the many mechanisms of dysbiosis-induced immunometabolic dysregulation, a decrease in circulating short-chain fatty acids (SCFAs), such as butyrate—a metabolic byproduct of intestinal microbes—limits their uptake by CD8^+^ T cells, thereby impairing their FAO-supported OXPHOS capability [48]. Consequently, CD8^+^ T cell commitment is skewed towards a terminal effector trajectory instead of memory differentiation upon antigen stimulation [49]. Dysbiosis induces an exhausted phenotype and diminished IFNγ responses in CD4⁺ and CD8⁺ T cells, promoting colitis-associated tumorigenesis. In eubiosis, pentanoate, an SCFA produced by gut microbes, inhibits class I histone deacetylases and enhances glycolysis through mTOR signaling, in turn increasing cytotoxic T lymphocyte effector molecules (including CD25, IFNγ, and TNF) and anti-tumor activity [50]; disrupting such microbiome balance compromises anti-tumor immunity.

In terms of dysbiosis-mediated metabolic rewiring of the innate immune system, microbiome imbalance favors hepatocellular carcinoma (HCC) development via IL-25-facilitated alternative activation of TAMs [51]. In particular, IL-25 promotes the preferential activation of anti-inflammatory macrophages via the upregulation of complex I, II, and III activity in the electron transport chain and its subsequent ROS-induced mitophagy [52,53]. Interestingly, the effects of dysbiosis on the immune system can be traced to as early in the differentiation pathway as bone marrow progenitor cells. In essence, dysbiosis-induced intestinal barrier permeability allows bacteria to migrate to the bone marrow, leading to expansion of the granulocyte–monocyte progenitor pool via Mincle-induced mTOR activation and glycolysis [54]; although providing an immune advantage in the response to pathogenic invasion, it can also support sustained low-grade systemic inflammation. Taken together, dysbiosis detrimentally compromises innate and adaptive immunity, whereby both arms of the immune system synergistically aggravate anti-tumor immunity, first by inducing chronic inflammation via myeloid activation, followed by consequent T cell exhaustion.

### Compromised physical and mental wellness

One of the less-explored means of immunomodulation is stress management. As psychological stress extensively affects neuroendocrine homeostasis, stress is then escalated to a systemic level [55]. In response to psychophysiological stress, glucocorticoid secretion into the circulation increases remarkably, likely via the hypothalamic–pituitary–adrenal (HPA) axis. In a model of sickle cell disease, a high glucocorticoid level effectively induced a “leaky gut” characterized by increased permeability of gut segmented filamentous bacteria through the lamina propria, triggering the polarization of T_H_17 cells and the subsequent secretion of IL-7α and G-CSF, on the one hand, creating a pro-inflammatory systemic environment, and on the other, expanding the pool of aged neutrophils [56]. Along the same line, high circulating levels of glucocorticoids are indicative of high levels of glucocorticoid receptor signaling; this activation has also been observed in neutrophils, where it signals the early diurnal aging of circulating neutrophils, which are highly susceptible to damage from ROS production and NETosis, thereby aggravating tumor metastasis [57]. In addition to glucocorticoid signaling, stress-induced neuroinflammation is accompanied by systemic upregulation of type I interferon [58], which can stimulate chronic systemic inflammation [59]. In the context of a tumor, such signaling can direct the differentiation of CD8^+^ T cells into a terminally exhaustive state [59]. Taking tumor immunity as an example to address the effect of stress on lymphocytes, the stress hormones catecholamines act through β-adrenergic receptors to impair CD8^+^ T cell activation by downregulating GLUT1 expression, in turn limiting their glucose uptake and glycolysis [60]. CD8^+^ T cell exhaustion has also been reported along these lines, whereby catecholamines interact with T cell ADRB1, an adrenergic receptor prominently upregulated in exhausted T cells, acting as a feedforward mechanism to sustainably activate cAMP activity and further promote T cell exhaustion [61]. Taken together, these findings point to the importance of addressing not only systemic oxidative stress but also psychological stress to improve immune fitness for better overall disease prevention and management.

### Aging and chronic low-grade inflammation

The role of aging in accelerating numerous aspects of disease has been extensively studied over the past few decades. Aging is a natural biological process that disrupts systemic metabolism and is marked by genomic instability, telomere attrition, epigenetic changes, and mitochondrial dysfunction [62]. In particular, cancers affecting adults over the age of 65 account for approximately 50% of all common cancers diagnosed [63]; hence, considering anti-aging approaches in prophylactic and therapeutic regimens may be helpful in cancer management. Linking back to immunometabolism, aged cells present with a senescence-associated secretory phenotype (SASP), which promotes chronic low-grade inflammation and senescence in immune cells (immunosenescence), thereby altering immunometabolic fitness and anti-tumor immunity [64]. In CD8^+^ T cells, age-related stress drives the accumulation of mitochondrial C14/C16-ceramide, which subsequently supports mitochondrial fission and mitophagy via PKA inhibition-related Drp1 upregulation; ultimately, this dampens CD8^+^ T cells’ oxygen consumption rate and activation [65]. Likewise, CD4^+^ T cells from older individuals are affected by ceramide-driven metabolic dysfunction. Acid sphingomyelinase, a lipid hydrolase that produces ceramide upon interaction with CD39 and CD161 on CD4^+^ T cells, is detectable at high levels in serum from older individuals [66]; the increased intracellular ceramide further signals STAT3 and mTOR activation and prompts T_H_17 cell polarization, ultimately leading to chronic low-grade inflammation [67]. In addition, the SASP significantly increases CD38 expression in macrophages, resulting in a decrease in the NAD pool and increased activation of the NAM salvage pathway as a compensatory mechanism [68]. These events sustain aerobic glycolysis and pro-inflammatory polarization of macrophages, ultimately leading to chronic systemic inflammation. Taken together, tackling aging and cellular longevity may provide an additional advantage for therapeutic efficacy, especially in cancers.

## Immunometabolic modulation in cancer therapeutic strategies

**Fig 3:**
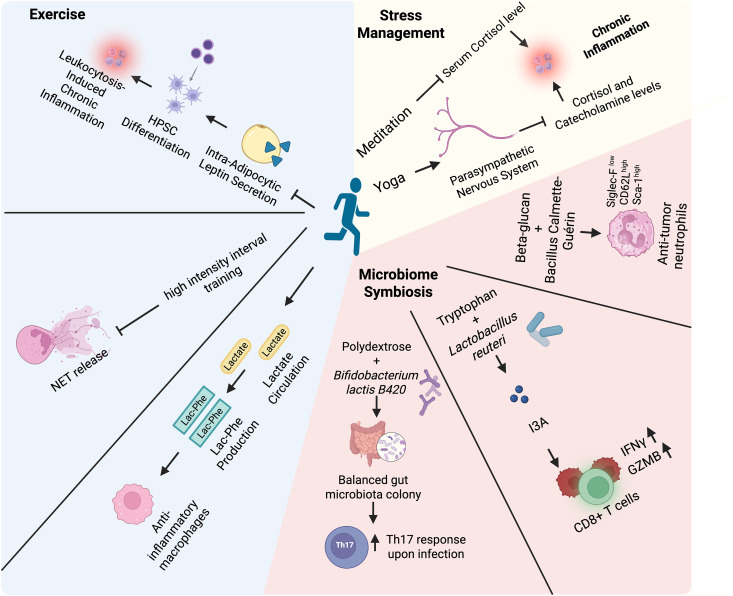
The role of physical and mental wellbeing in restoring immunometabolic fitness. A sedentary lifestyle is detrimental to immunometabolic fitness. Physical exercise impacts immune cell metabolism by decreasing intra-adipocytic leptin secretion, subsequently reducing myeloid HPSC differentiation and leukocytosis-induced chronic inflammation. High-intensity interval training reduces NET release by human neutrophils and exercise-induced increased lactate enables Lac-Phe production and polarizes macrophages to an anti-inflammatory phenotype. Maintaining microbiome symbiosis by using beta-glucan and the BCG vaccine as a synbiotic promotes the development of anti-tumor neutrophils. Co-administration of tryptophan and *Lactobacillus reuteri* pivots tryptophan to produce I3A, which favors CD8^+^ T cell effector function and cytokine secretion (IFNγ and GZMb). Polydextrose and *Bifidobacterium lactis* B420 can reshape a balanced gut microbiota colony to one which favors T_H_17 cell responses upon infection. Stress management using techniques such as meditation and yoga could activate the parasympathetic nervous system and reduce cortisol and catecholamine levels, resulting in overall amelioration of chronic inflammation. *Created in BioRender. Tan,* R. *(2026)* *https://BioRender.com/n4tfydb*.

As we have already discussed, both cellular and systemic metabolism respond to intrinsic and extrinsic factors, and a shift in either may result in metabolic dysregulation and give rise to various metabolic diseases. In cancer, although its initiation is a result of uncontrolled proliferation, the presence of a tumor dynamically changes systemic metabolism; over time, these changes can promote tumor progression, immune evasion, and even metastasis. To date, there are only a few drugs on the market that target cellular metabolism that are licensed to treat metabolic diseases (Table 1). These drugs also reportedly modulate immunometabolism, which although an off-target effect, enables us to explore the possibilities of incorporating such metabolic drugs into therapeutic regimes with the aim of rewiring immunometabolism and for treating a broader spectrum of diseases. While pharmacological intervention can be used to correct cell intrinsic defects, extrinsic metabolic rectification depends on physical and psychological interventions, such as diet, exercise, mental health improvement, and anti-aging approaches (Fig 3).

**Table 1:** Examples of US FDA-approved drugs that possess immunomodulatory effects.

US FDA-approved drugs that affect metabolism	Functions in tumor immunometabolism	References
Metformin	CD8^+^ T cells: ↓ mitochondrial complex I activity, ↓ ROS accumulation, ↓ mitochondrial dysfunction, and oxidative DNA damage, ↑ IFNγ and TNF expression, ↑ proliferative ability, ↓ PD-1 and LAG-3 expression	[69,70]
	Macrophages: ↓ OXPHOS, ↑ glycolysis, ↑ IL-12 and TNF production, ↓ IL-10 production, ↑ MHC II expression, ↑ pro-inflammatory polarization	[71]
	Neutrophils: ↓ NADPH oxidase, ↓ ROS production, ↓ NET production	[72,73]
Simvastatin	T cells: ↑ FOXP3^+^ T_reg_ cells, ↑ CD8^+^ T cell activation	[74,75]
Disulfiram	CD8^+^ T cells: ↑ glycolysis and OXPHOS, ↑ T cell activation, ↑ IL-2, IFNγ, and TNF production, ↑ proliferative ability	[76]
Exendin-4	T cells: ↓ FOXP3^+^ T_reg_ cells, ↑ IFNγ-producing CD8^+^ T cells	[77]

### Dietary and nutritional interventions to improve immunometabolism

#### Dietary nutrients and supplements.

Dietary metabolites support and maintain cellular functions in physiology and in pathology. Taking solid tumors as an example, cancer cells often out-compete their immune counterparts in the TME when it comes to energy utilization, supporting their survival and proliferative needs while creating a fuel desert for infiltrating leukocytes, thereby compromising the functionality of the leukocytes and increasing their susceptibility to pro-tumorigenic reprogramming [78]. In addition, some metabolites are preferentially taken up by immune cells in the TME to sustain their pro- or anti-inflammatory activities [79]. Hence, understanding the metabolic architecture of each cell type, particularly of immune cells, is instrumental to optimizing dietary schemes for better disease management. Several dietary interventions have been suggested for a healthier lifestyle; namely, a galactose-rich diet to strengthen anti-tumor immunity [80], succinate supplementation to ameliorate inflammation [81], tryptophan restriction, methionine supplementation, and a ketogenic diet. As the former two dietary interventions are mostly discussed in relation to effects on non-immune cells, we will focus more on the latter three approaches in this Essay.

Tryptophan metabolism in immune cells, per se, has long been thought to be immunosuppressive [82]. Canonically in CD8^+^ T cells, the serotonin pathway is involved in tryptophan catabolism. In essence, extracellular IL-2 triggers the transcription of *Thp1* via STAT5 activation and allows THP1 conversion of tryptophan into 5-HT, a precursor of serotonin. High intracellular levels of 5-HT facilitates AhR nuclear translocation, whereby it promotes sustained T cell activation. However, persistent activation of CD8^+^ T cells drives their exhaustion, resulting in an immunosuppressive phenotype [83]. In glioblastoma, AhR activation in TAMs is heightened in response to kynurenine, a metabolic byproduct of tryptophan metabolism [84]. This hyperactivation drives a series of synergistic effects contributing to tumor immune evasion; first by inducing CCR2 expression on TAMs, resulting in TAM retention in the TME, meanwhile upregulating KLF4 to allow pro-tumorigenic reprogramming of TAMs into a CD39^hi^ subset. Consequently, CD39 and CD73 synergistically catalyze extracellular ATP-derived adenosine release; upon uptake into CD8^+^ T cells, it hampers de novo pyrimidine synthesis and dampens their functionality, as well as their cytokine-producing capability [85]. These studies suggest that limiting tryptophan availability may shape an anti-inflammatory immune profile. Indeed, low tryptophan availability enhances ICB responses in a pre-clinical model of HCC [86]; low intra-tumoral tryptophan-derived kynurenine favors tertiary lymphoid structure maturation via germinal center B cell formation, allowing an enhanced niche function in the spleen. Indeed, dietary tryptophan restriction can be used as a way of promoting immune functionality, with the hope of improving disease management in the context of cancer, yet long-term tryptophan deprivation may trigger compensatory responses along the lines of cancer adaptation; hence, it is important to tailor nutritional strategies according to an individual’s metabolism, as well as cancer types, in a clinical setting.

Methionine has an important role in immune surveillance, especially in T cell metabolism and functionality [87,88]. For example, T cells utilize methionine to counteract NFAT1 activation, which drives T cell exhaustion by overburdening of Ca^2+^ influx-induced TCR activation [89]. Methionine supplementation allows its preferential uptake by CD4^+^ T cells rather than by tumor cells, in turn promoting AMPK activation of CD4^+^ T cells and restricting PD-1 expression to strengthen anti-tumor immunity and restrict tumor growth in B16 melanoma [90]. In addition, dietary methionine deficiency leads to reduced polarization of T cells into a memory phenotype in a preclinical model of colorectal cancer; an imbalanced *Firmicutes/Bacteriodetes* ratio in tumor-bearing mice resulted in intra-tumoral sulfur deficiency and ultimately aggravated tumor burden with a poorer anti-PD1 response [91]. Methionine is evidently essential for supporting T cell anabolism, especially in the context of tumor immunity, yet a complete enrichment of methionine in the daily diet may risk activation of compensatory mechanisms in other cell types. Therefore, making use of a tailored dietary scheme according to individual clinical parameters and microbiota composition may be more effective in achieving an optimal ICB therapeutic outcome.

The ketogenic diet, a high-fat, low-carbohydrate regimen, has gained attention for its immunomodulatory effects. It is formulated to trigger hepatic ketogenesis, whereby ketolytic immune cells can utilize ketone bodies to replenish their energetic needs in an environment of low glucose availability [92,93]. Inevitably, glucose is essential to mount an immune response as it is the primary fuel for glycolysis, which is the pillar for cell activation [94,95]. However, in solid tumors, the Warburg effect depletes glucose, creating a nutrient-poor TME for immune cells [96]. The concept of a ketogenic diet is to simultaneously insulate tumor cells from glucose accessibility while providing an alternative source of nutrients for effective immune cell activation, ultimately accelerating cancer regression from tumor-intrinsic and extrinsic approaches. In T cells, increased availability of ketone drives ketolysis and triggers aerobic oxidation via NLRP3 suppression to improve mitochondrial fitness, resulting in memory T cell formation and enhanced overall functionality [97]. Ketolysis-improved T cell functionality is preserved by increasing the release of acetyl-CoA in a feedforward manner, which further fuels the TCA cycle for optimal CD8^+^ T cell activation and effector function [98,99]. The same study showed that ketone uptake by CD8^+^ T cells remains prominent in the nutrient-deprived TME and that tumor growth is stunted in a pre-clinical model of colorectal cancer.

In terms of cancer management, a ketogenic diet directly facilitates proliferation, activation, and antigen presentation of Nos2^+^ dendritic cells, but not other subsets, in the TME via β-hydroxybutyrate (BHB)-induced NLPR3 activation, consequently chemoattracting CD8^+^ T cells through CXCR3 signaling for better a ICB outcome in a mouse model of prostate cancer [100]. The anti-tumor effect of a ketogenic diet has also been reported in other cancer types. In particular, a ketogenic diet enables an increase in the circulating ketone body BHB, which facilitates mitochondrial respiration in immune cells and limits their glucose dependence, reconstructing immune fitness to a less exhausted CD4^+^ and CD8^+^ T cell phenotype, as well as producing less immunosuppressive polarization of myeloid cells [101]. In synergy, this suppressed tumor growth and enhanced ICB responses in melanoma and renal cancer models [101]. As previously mentioned, chronic systemic inflammation has a detrimental role by dampening anti-tumor immunity. In a non-tumor context, BHB downregulates NLRP3 signaling in circulating neutrophils and in TRMs, reducing caspase-1 activity and subsequent secretion of IL-1β, eliciting an anti-inflammatory state [102]. This BHB-induced NLRP3 downregulation was due to stabilization of the intracellular potassium pool, whereby heightened potassium efflux is a well-known activator of NLRP3 inflammasome [103]. Taken together, a ketogenic diet could be adopted into routine cancer treatment schemes, first to rectify systemic low-grade inflammation, then to rejuvenate activity of tumor infiltrating immune cells, finally leading to enhanced disease regression; however, considering the high fat content of such a dietary regimen, it is also important that healthcare professionals take the risks of developing other co-morbidities, such as hypertension and elevated circulating cholesterol, into account when giving dietary advice, especially to older patients.

#### Intermittent fasting.

Intermittent fasting (IF), defined by feed–fast oscillation with usual calorie intake, is used as a weight loss strategy [104–106]. In recent years, it has been further explored as an immune-reprogramming approach to enhance metabolic fitness. Of note, IF lowers systemic inflammation via redistribution of immune cells, pre- and post-fasting. For instance, in response to low energy levels, AMPK signaling in hepatocytes is activated, affecting glycolysis and creating a dormant state in bone marrow monocytes; accompanied by a reduction in plasma CCL2 and monocyte CCR2, monocyte egress is halted [107]. In addition, neuroimmune communication changes in response to fasting, with phenotypically quiescent T cells redistributed to the bone marrow upon catecholaminergic neuronal activation in the ventrolateral medulla via the CXCR4–CXCL12 axis [108]. Current evidence therefore suggests that systemic inflammation can be maintained at low levels through IF, potentially achieving improved physiological wellbeing.

Exploring the therapeutic benefits of IF, several studies have been conducted to utilize IF in the context of anti-tumor immunity. In pre-clinical models of epithelial ovarian cancer, IF effectively enhanced ketogenesis and BHB levels in T cells, resulting in an enhanced anti-PD-1 response [109]. A link between BHB and T cells has been established, whereby BHB allows the metabolic conversion from glycolysis to mitochondrial dependence in CD4^+^ T cells, as well as an increase in FAO ability [110]; although this study was conducted in the context of viral infection, improved T cell plasticity may suggest better adaptation in the TME with the presence of BHB. In addition, glucocorticoid signaling crosstalk has been described between macrophages and hepatocytes in response to IF. In essence, glucocorticoid receptor upregulation in macrophages upon IF suppresses TNF secretion, which in turn triggers ketogenesis in hepatocytes and the subsequent release of ketone bodies [111]; as previously mentioned, ketolytic immune cells could benefit from this for sustained energetic needs. In conjunction with this, IF protects the mice from developing diet-induced metabolic dysfunction-associated steatohepatitis (MASH)-related HCC via glucocorticoid-induced activation of PPARα and PCK1 in hepatocytes [112]. Given that MASH-related HCC is enriched with highly pro-tumorigenic SiglecF^+^ TANs [113], it is sensible to suggest that IF-ameliorated MASH-related HCC may foster an unfavorable TME for TAN development. Indeed, it is known that low glucose availability triggers the secretion of cortisol via the HPA axis as a stress response mechanism [114], and that elevated cortisol correlates with poor immune activation and function [115]. With regards to this, glucose deprivation-triggered hepato-ketogenesis is capable of reversing the resultant T cell functional defect via BHB metabolism [116]; therefore, although the effect of acute glucose starvation on immune function is negligible, individual response to IF alongside cancer treatment should still be monitored and optimized. IF also fosters a glucose-deprived circulation, which starves tumor cells of glucose, making it a dual-targeted approach to improve cancer treatment outcomes. However, as IF is voluntary and physiologically untraceable, strict patient adherence to such a lifestyle change would be essential; nevertheless, IF can be encouraged as a lifestyle adaptation for patients with cancer who wish to try and enhance their treatment outcomes.

#### Calorie restriction.

Another dietary intervention that can rewire biological metabolism, widely used in weight management and studied in life span research, is calorie restriction (CR) [117–119]. Not only does CR modulate tissue homeostasis [120–122], it also regulates immunometabolism [123]. As the name suggests, CR involves limiting daily calorific intake without causing malnutrition [119]. One possible mechanism by which CR dictates immune fitness is via amelioration of chronic systemic inflammation [124]. In particular, a 14% reduction in daily calorie intake diminished inflammasome activation in ATMs, restoring homeostatic mitophagy in macrophages [125] and resulting in an overall decrease in pro-inflammatory cytokine secretion upon ceramide stimulation [124]. A clinical study revealed that CR at 825–853 kcal daily for 5 months lowered HbA1c in patients with T2D, resulting in T2D remission [126]; high HbA1c correlates with diminished T_H_1 cell polarization and glycolytic functionality via increased LPO in CD4^+^ T cells [36]. Therefore, lifestyle adoption of CR not only reduces macrophage-mediated chronic inflammation but also enhances T_H_1 cell immunity via restoration of optimal glycemic control.

Chronic inflammation and heightened HbA1c levels are known risk factors for tumor development and correlate with dampened ICB responsiveness [25,127]; as CR could potentially alleviate these pathophysiological manifestations, its application in cancer management is highly encouraged. As previously mentioned, T_H_1 cell functionality can be restored by CR; besides anti-viral immunity, T_H_1 cells are also instrumental in regulating anti-cancer immunity. T_H_1 cells in the TME prime TAMs to secrete CXCL9 and CXCL10 (chemoattractants of CD8^+^ T cells) under IFNγ stimulation, in turn improving ICB responses [128]. Phenotypically, CR systemically reduces regulatory T cells while promoting the infiltration of effector and memory T cells into the TME in breast cancer models, resulting in retarded tumor progression and metastatic capacity [129]. Moreover, enhanced immunosurveillance upon CR was observed in mouse model of colorectal cancer, whereby *Bifidobacterium* dominance in the gut elevated acetate production, circulation, and migration into the TME, resulting in subsequent infiltration of IFNγ^+^ CD8^+^ T cells [130]. In summary, CR is a promising intervention to mitigate anti-tumor immunity and ICB responsiveness.

### Promoting physical and mental wellbeing to regain metabolic balance

#### Exercise.

Systemic inflammation has been extensively shown to be a cause of aging [131] and cancer progression [132], while exercise seems to be one of many rescue factors (Fig 3). The effect of exercise on physiology occurs via a series of secretomic shifts; one such well-established shift is a decrease in intra-adipocytic leptin secretion [133,134]. Consequently, such a reduction in circulating leptin skews hematopoietic stem and progenitor cell (HSPC) metabolism towards OXPHOS, halts their differentiation, and strengthens their confinement to the bone marrow via CXCL12–CXCR4 activity [135]; leukocytosis-induced chronic inflammation can then be prevented. Indeed, chronic inflammation and adiposity are highly interlinked, and the resultant effects on immune cells are apparent. Feng and colleagues have postulated that adipocyte-derived lactate local accumulation potentially stabilizes HIF-1α in TRM via selective binding to the lactate sensor prolyl hydroxylase domain 2, promoting TRM IL-1β secretion and eventually metaflammation [136]. As opposed to local accumulation of lactate, circulating lactate serves as a source for N-lactoyl-phenylalanine (Lac-Phe) production, an exercise-induced switch towards macrophage lactate and phenylalanine anabolism via CNDP2. In addition, a positive correlation exists between plasma Lac-Phe levels and anti-inflammatory polarization of macrophages via the suppression of the p65–NFκB cascade and its resultant decrease in IL-1β, IL-6, and TNF secretion [137]. These studies suggest an autocrine and positive feedback mechanism of Lac-Phe in modulating macrophage reprogramming, consolidating the significant role of Lac-Phe in exercise-mediated inflammation reduction.

In addition to macrophages, neutrophils are also important in exercise-induced anti-inflammatory effects. High-intensity interval training decreases NET release upon NETosis, especially in older individuals [138] (NETosis is a well-known consequence of oxidative stress). In the context of tumor biology, NETosis is not only pro-tumorigenic but also pro-metastatic. Mechanistically, an increase in NETosis by Ly6G^hi^Ly6C^lo^ neutrophils, which are highly retained in the blood vessels [139], results in vascular occlusion and tumor necrosis, leading the tumor to start to metastasize for nutrition. Of note, a role for exercise in ICB responses has been reported, whereby the production of formate by gut microbes upon exercise-induced 1-carbon metabolism promotes Nrf2–IRF8–STAT1 activation in CD8^+^ T cells, further enhancing their functionality, antigen specificity, and pro-inflammatory cytokine production to boost ICB response in melanoma [140]. Exercise also promotes T cell antigen-specific functionality via formate-induced Nrf2 activation and subsequent intracellular anti-oxidation, leading to ICB efficacy in a pre-clinical model of melanoma [141]. Altogether, although the metabolic mechanism of exercise-induced manifestation has been less-well explored, the overall anti-inflammatory effect of exercise indicates that the incorporation of exercise into daily routines to enhance ICB responses is promising.

#### Stress management and parasympathetic nervous response.

In a previous section, we described the relationship between psychophysiological stress and systemic chronic low-grade inflammation. Along with many efforts to promote mental health awareness to mitigate metaflammation, the importance of mindfulness in physiological health has been emphasized. Indeed, patients with non-small cell lung cancer with high levels of emotional distress upon diagnosis exhibited poorer ICB prognosis, as well as lower progress-free survival, overall survival, and quality of life than those with low levels of emotional distress [142]; the serum level of cortisol was also elevated in emotionally distressed patients. This finding consolidated the role of mental wellbeing in dictating cancer treatment outcomes and suggested that glucocorticoid signaling may be a key cause of the observed response. Notably, in a trial conducted on stressed young adults, a provisional mindfulness meditation significantly lowered their serum cortisol levels by approximately 20% [143]. Yoga, although physical, is also considered a mindfulness approach as it activates the parasympathetic nervous system, indicating relaxation. Yoga practice can effectively lower depression, anxiety, psychosis and obsession scores, along with reducing cortisol and catecholamine levels [144]. In conjunction with studies showing that increased glucocorticoid receptor and catecholamine signaling correlate with heightened cancer burden, these findings could further support the notion that mindfulness can be an integrative effort to enhance anti-tumor immunity, especially in ameliorating neutrophil and T cell metabolism-induced chronic inflammation.

#### Symbiosis of the microbiome.

Instead of directly affecting immune cells, the gut microbiome serves as an important regulator of nutrients that are fed into the immune system. Of note, the gut microbiome can be particularly sensitive to environmental changes, and dysbiosis often follows chronic systemic inflammation. For instance, consumption of a high-fat diet by mice resulted in a microbiome that was different from that of chow-fed controls. By synbiotic (Box 1) treatment of these obese mice with polydextrose and *Bifidobacterium lactis B420*, the high-fat diet-induced microbiome shift was rescued, followed by further reshaping of the microbiome to a high *Sutterellaceae*, *Coriobacteriaceae*, and *Bacteroidaceae*, low *Lachnospiraceae* colonizing gut, improving intestinal T_H_17 cell response against infection [145]. Although a systemic increase in T_H_17 cells was attributed to chronic inflammation, this study showed that T_H_17 cells and regulatory T cells are both reduced upon high-fat diet consumption, suggesting that a balanced pro- and anti-inflammatory cell pool is more important in the context of metabolic diseases.

Moreover, multiple studies have reported on the promising effect of synbiotic treatment in the context of cancer management. For example, dietary tryptophan is canonically a pro-tumor factor, whereas the co-administration of *Lactobacillus reuteri* as a probiotic can effectively ameliorate this effect [146]. This result was associated with the secretion of indole-3-carbaldehyde (I3A), a tryptophan catabolite, which activates tumor-infiltrating CD8^+^ T cells and improves their cytotoxicity via AhR signaling; ICB responses in a preclinical model of melanoma were only improved when tryptophan and *Lactobacillus reuteri* coexisted temporally and spatially, suggesting that I3A-driven tryptophan catabolism may reverse the pro-tumor effect of tryptophan [146]. Patients with colorectal cancer reportedly experienced better disease management and radio-chemotherapy response upon ketogenic diet switch [147]; further experiments in mice with a humanized microbiome showed that a ketogenic diet restructured a high stearic acid-producing microbiome, which acts as an effective apoptosis inducer of tumor cells and a colonic T_H_17 cell suppressor [148]. Furthermore, the administration of *Lactobacillus plantarum* L168 tailored a gut microbiome with high indole-3-lactic acid levels that effectively inhibited serum amyloid A3-induced high-density lipoprotein retention in CD8^+^ T lymphocytes [149]. The resultant lowering of intracellular cholesterol allowed better T cell activation, as well as IFNγ and GZMB production, ultimately leading to a retardation of tumor growth. Simultaneously, the authors also discovered a lowered circulating level of pro-inflammatory IL-1β, IL-6, and IL-17α, along with an increased level of anti-inflammatory IL-12, IFNγ, and IL-10 in the context of colitis-associated cancer, suggesting ameliorated systemic inflammation [149].

In the context of synbiotic cancer vaccines, parallel administration of beta-glucan (as a prebiotic; Box 1) and BCG (as a probiotic; Box 1) synergistically trained HSPCs for better granulopoiesis of matured neutrophils [150]. Unlike classic neutrophils, which are pro-tumor upon tumor infiltration, these neutrophils are long-lived and are less pro-tumorigenic, characterized by low dcTRAILr1 and SiglecF, and high CD62L expression. The authors of this study further reasoned that neutrophil resistance to reprogramming may be due to impaired tumor vasculature. This synbiotic treatment has been shown to improve ICB sensitivity in bladder cancer and melanoma in mice [150]. Although most studies have reported positive outcomes for synbiotic and probiotic treatments, especially in the context of cancer, much remains to be discovered as the complexity of the gut microbiome and the dynamic evolution of cancer complicate the concept; nevertheless, regulating the gut microbiome is a very promising option as a complementary treatment regimen to improve ICB responses.

### Slowing aging to regain immune metabolic fitness

Aging, an inevitable and evolutionarily conserved process of physiology, contributes to various types of age-related metabolic pathophysiology [151]. Inflammaging is highly associated with immunosenescence, whereby senescent immune cells secrete SASP, further causing suboptimal functionality of the immune system in a positive feedback manner [64]. Therefore, senotherapeutics (Box 1) could be used to mitigate inflammaging and thereby manipulate the metabolic functionality of immune cells. Indeed, older animals have an increase in T_H_1 and T_H_17 cells caused by hyperactivation of dendritic cells. This hyperactivation has been attributed to an increase in cell-free mitochondrial DNA expelled by senescent cells, with senolytic treatment with dasatinib and quercetin effectively reversing pro-inflammatory shifts, characterized by decreased IL-17 and IFNγ production in CD4^+^ T cells [152]. Another study also reported similar findings, showing that the plasma levels of T_H_17 cell-mediating cytokines, namely IL-16, IL-17A, IL-17C, IL-21, and MIP-3α, were reduced upon senolytic treatment in older mice [153]. Additionally, senescence-induced aging is driven by increased myelopoiesis in dispense of lymphopoiesis, ultimately disrupting the homeostatic response of TRMs when monocyte infiltration dominates in response to infection [154]. In accordance with this, the BCL-2 inhibitor ABT263 effectively rebalanced a myeloid-skewed hematopoiesis profile, potentially through the specific clearance of senescent stem cells in the bone marrow [155]. Notably, several senolytic compounds aiming to restore apoptotic mechanisms via BCL-2, BCL-XL, and BCL-W inhibition in cancer cells are currently undergoing clinical trials, highlighting their potential as adjuvants in cancer management [156]. Collectively, the effect of senolytics in reversing systemic low-grade inflammation is conspicuous, as is their effect in reducing chronic inflammation to improve cancer treatment outcome; however, less has been reported about their effect on immunometabolic rewiring. Nevertheless, this evidence provides an exploratory framework to further expand the use of senotherapeutics not only in cancer treatment and prevention, but also in other inflammatory, metabolic diseases, such as T2D and obesity.

## Future directions and conclusion

Diseases of all kinds influence systemic metabolism; likewise, systemic metabolism modulates immunometabolism and ultimately dictates immune function and fate decisions. Energy use and expenditure are leniently governed, being both essential for homeostatic regulation but also easily manipulated in response to environmental changes. Especially in diseases such as cancer, where dysfunction of one signal leads to another, forming a feedforward loop of metabolic dysregulation, efforts to achieve a healthier systemic metabolism are of the utmost importance to try and regain homeostatic balance.

A healthy lifestyle can reshape cellular and systemic metabolism, complementing pharmacological interventions. Although medical interventions have been developed to tackle metabolic diseases such as T2D and hypercholesterolemia, many patients still fail to achieve optimal disease control, and can even expose themselves to risks of developing malignancy. Therefore, from a prophylactic and treatment point of view, dietary lifestyle intervention could be incorporated into therapeutic strategies to amplify beneficial outcomes. For instance, the T2D drug metformin is able to reduce NETosis and neutrophil ROS production (Table 1). This is also the case of stress signaling inhibition, whereby blunted glucocorticoid receptor signaling results in reduced NETosis and controlled metastasis in mice [57]. In conjunction with the findings that mindfulness practices can reduce circulating glucocorticoids [143], the combination of practicing mindfulness and taking metformin could therefore potentially synergistically attenuate glucocorticoid receptor signaling and prevent subsequent NETosis-mediated metastasis in patients at higher metastatic risk. In addition, most of the drugs listed in Table 1 reduce exhaustion in CD8^+^ T cells, hinting at their ability to rejuvenate intra-tumoral CD8^+^ T cells; by adopting a methionine-restricted and ketogenic diet in combination with these metabolism-regulating drugs, anti-tumor responses could potentially be enhanced upon ICB therapy in patients with cancer.

While most of the described efforts are aimed at reducing systemic inflammation, such practices can also be incorporated into the lifestyle of patients with cancer to enhance therapeutic efficacy by restoring immunometabolic fitness. As a proof-of-concept, several studies have demonstrated that a ketogenic diet or its derivatives improve ICB responses in mouse models via an effective reduction in the exhausted T cell pool [100,101,157]. These lifestyle habits may also benefit the treatment of metabolic diseases such as T2D and obesity in a way that lowers the risk of patients developing additional co-morbidities or risk of fatality when encountering pathogenic infections. Senolytics could also be considered in older patients to lower systemic inflammation, in the hope of obtaining better cancer prognosis, as they have been demonstrated in mouse model of colorectal cancer to produce an overall reduction in immunosuppressive monocyte accumulation in the TME [158].

Appropriate support in encouraging a healthy lifestyle, both physically and mentally, should therefore be encouraged to improve therapeutic outcomes for individuals with cancer and other metabolic diseases, although personalized therapeutic strategies will need to be considered for optimum disease management. By tackling metabolism from a two-pronged view, we believe that an improved lifestyle via diet, exercise, mindfulness, anti-aging and microbiome-modulating intervention could effectively alleviate metabolic and inflammatory stresses to the body, in turn enabling full immune functionality to fight diseases.

## Abbreviations

ATMs: adipose tissue macrophages
BaP: benzo[a]pyrene
BHB: β-hydroxybutyrate
FAO: fatty acid oxidation
HCC: hepatocellular carcinoma
HPA: hypothalamic–pituitary–adrenal
HSPC: hematopoietic stem and progenitor cell
I3A: indole-3-carbaldehyde
ICB: immune checkpoint blockade
IF: Intermittent fasting
LPO: lipid peroxidation
MASH: metabolic dysfunction-associated steatohepatitis
NNK: Nicotine-derived nitrosamine ketone
OXPHOS: oxidative phosphorylation
PPP: pentose phosphate pathway
ROS: reactive oxygen species
SASP: senescence-associated secretory phenotype
SCFAs: short-chain fatty acids
T2D: type 2 diabetes
TAMs: tumor-associated macrophages
TANs: tumor-associated neutrophils
TCA: tricarboxylic acid
TCR: T cell receptor
TME: tumor microenvironment
TRMs: tissue-resident macrophages
